# The effect of road and sea transport on inflammatory, adrenocortical, metabolic and behavioural responses of weanling heifers

**DOI:** 10.1186/1746-6148-6-36

**Published:** 2010-07-20

**Authors:** Bernadette Earley, Margaret Murray

**Affiliations:** 1 Animal and Bioscience Research Department, Animal & Grassland Research and Innovation Centre, Teagasc, Grange, Dunsany, Co. Meath, Ireland

## Abstract

The objective was to investigate the effect of transport on inflammatory, adrenocortical, metabolic and behavioural responses of weanling heifers transported from Ireland to Spain.

**Background:**

At the end of the grazing season, 60 Charolais × beef heifers (mean live weight 245, s.e. 4.3 kg and mean weaning age 219, s.e. 4.9 days) were abruptly weaned from their dams on day 0. The animals were assigned by live weight to two treatments, transport (T) (n = 40) (mean 246, s.e. 5.4 kg) and control (C) (n = 20) (mean 247, s.e. 7.2 kg) on day 0. The T animals were transported from Ireland to France on a roll-on roll-off ferry at a stocking density of 0.93 m^2^/animal and then by road for 9 h to a French lairage while C animals remained in Ireland and were not transported. On arrival at the French lairage (d 2), 20 T animals were unloaded (ULT) and rested for 12 h in the French lairage and 20 animals rested (RT) on the transporter. All animals had access to hay and water. After the rest period, the ULT animals were re-loaded. The subsequent journey by road from France to Spain was 9 h travel, 7 h rest (on the transporter) and a further 7 h travel by road. All T animals were blood sampled prior to transport (day (d) 0; baseline), on arrival in the French lairage (d 2), after 12 h rest in the French lairage (d 2), on arrival at the feedlot in Spain (d 4) and on d 6, d 8, d 10 and d 34. Control animals were blood sampled at the same times as T animals.

**Results:**

ULT and RT animals had lower (P < 0.05) live weight than C animals on d 6. WBC number was lower (P < 0.05) in control animals on d 10 and greater on d 34 compared with baseline values. RT and ULT animals had greater (P < 0.05) WBC number than baseline on d 2 (arrival in France) to d 34. Neutrophil % was unchanged in RT, ULT and control animals compared with baseline. Control, RT and ULT animals had lower haematocrit % (P < 0.05) throughout the study compared with d 0. There was no difference (P > 0.05) in plasma protein concentration between RT and ULT animals from day 2 to d 34. Plasma concentrations of urea were higher (P < 0.05) in RT animals from d 2 to d 34 compared with C animals. RT and ULT animals had lower (P < 0.05) non-esterified fatty acid (NEFA) and βeta-hydroxy-butyrate (ßHB) concentrations on d 10 and d 34 compared with d 0.

**Conclusion:**

It is concluded that, within the conditions of the present study, the performance of the animals that remained on the transporter during the 12 h lairage period in France was not different post-transport from the transported animals that were unloaded and lairaged in France.

## Background

The protection of animals during transport is an important animal welfare concern. An on-going revision of EU laws in relation to animal transport is taking place and more objective scientific evidence is needed in order to inform policy makers. In July 2003, the European Commission adopted a proposal for a Regulation on animal transport which sought to radically overhaul the rules governing the transport of animals in Europe for journeys of more than 8 h, including domestic transport in Ireland and long duration journeys to the Continent. There have been few studies that have investigated the effects of transport of weanling heifers in a Roll-on Roll-off (RO-RO) vessel by sea, followed by road transport, with a mid-journey lairage of 12 h duration on the transporter, on the inflammatory, adrenocortical, metabolic and behavioural responses of animals before and after transport. In addition, no studies have investigated the effects on the animals' physiological responses if they are allowed to rest on a transporter without unloading following a sea journey (23 h) and land transport (9 h). Studies have investigated the physiological and behavioural consequences of the transport of heifers, bulls and steers by road from northern Germany to Mediterranean ports and concluded that animals should be prepared carefully pre-transport, i.e. with reference to energy and fluid balance, and to be fed at sufficient time intervals during the journey to maintain physiological homeostasis and normal expression of behaviour [1]. In another study [2], the effects of space allowance during transportation and duration of a mid-journey lairage period on measures of stress, injury, dehydration, food restriction and rest in young calves were investigated. The authors concluded that the duration of the mid-journey lairage was not an important factor and while there was little evidence that transport affected immunological variables, there was evidence to indicate the health of the calves was adversely affected post transport. The confinement of animals on a moving vehicle has been reported to be the most stressful component of transportation [3,4], while others have reported that loading and unloading cause the most stress to cattle [5]. Knowles [6] investigated the physiological and behavioural effects in cattle of transporting them for periods of 14, 21, 26 and 31 hours, including a stop for a rest and drink on the lorry after 14 hours. The authors concluded that the physiological changes that were found after a 31 h journey indicated that transport was not detrimental to the animals. A review of the behavioural, physiological and immunological consequences of animal transport research, with relevance to the dairy industry, concluded that the duration of the journey has a greater impact than the distance travelled on young calves, and that after long transport, most animals drink and then rested. Studies also showed that young calves habituate to transport, unlike cows [7].

Physiological and haematological responses associated with transport of animals are well studied. Increased neutrophil and decreased lymphocyte numbers following transportation have been documented in previous works [8-13]. During transport, Grandin [14] suggested that animals become stressed by either psychological stress (restraint, handling, novelty) or physical stress (fasting, fatigue, injury or thermal extremes). During long distance transport animals are subjected to fasting periods and exhaustion [15,16]. Increased heart rate and plasma stress hormones (cortisol, adrenaline) during transport are a consequence of a non-specific stress reaction to the novelty of the transport process and the environment, which has been reported to elicit a wide array of physiological changes in blood constituents [17]. There is a growing interest in the measurement of acute phase proteins (APP) as indicators of the inflammatory process. Haptoglobin, an APP is released from hepatocytes in response to tissue injury or infection [18-20]. Plasma concentration of fibrinogen, another APP, has been shown to increase in cattle with inflammatory disorders [21] and was used for many years to evaluate inflammatory disease in cattle [22-24].

The objective of the present study was to investigate the effect of sea and road transport on inflammatory, adrenocortical, metabolic and behavioural responses of weanling heifers transported from Ireland to Spain. We tested the hypothesis that unloading cattle prior to lairage is beneficial to their welfare. To test this hypothesis and in accordance with routinely used physiological indicators of welfare [8,9] we examined physiological markers of stress (cortisol, creatine kinase), immunity (PHA-induced and Con-A induced interferon-γ), acute phase proteins (haptoglobin and fibrinogen) and haematological profiles. In relation to fear, arousal and physical activity we measured physiological markers of energy metabolism (βHB, albumin, protein, glucose, NEFA and urea) before and after transport. In addition, behaviour, live weight, and rectal temperature were monitored.

## Results

### Environment

The mean temperature of the ambient environment before transport was 10.0°C (± (s.e.) 0.09) and the mean temperature of the shed where the animals were assembled pre-transport was 12.9°C (± (s.e.) 0.18). The environmental data recorded during the ferry journey were; the mean CO_2 _concentration recorded was 828 ppm (± (s.e.) 17); mean relative humidity 73.4% (± (s.e.) 0.5); mean temperature 16.0°C (± (s.e.) 0.3); mean wind velocity 0.20 m/s (± (s.e.) 0.02); mean vapour density 11.2 g/m^3 ^(± (s.e.) 0.22). During the road journey from France to Spain the environmental data recorded were; mean CO_2 _concentration was 807 ppm (± (s.e.) 27.8); mean relative humidity 65.0% (± (s.e.) 0.90); mean temperature 13.4°C (± (s.e.) 0.3); mean wind velocity 0.40 m/s (± (s.e.) 0.04); mean vapour density 6.7 g/m^3 ^(± (s.e.) 0.19). The environmental data recorded while animals remained on the transporter in France for 12 h was; mean CO_2 _concentrations 874.8 ppm (± (s.e.) 46); mean relative humidity 75.7% (± (s.e.) 0.9); mean temperature 13.9°C (± (s.e.) 0.40); mean wind velocity 0.14 m/s (± (s.e.) 0.03); mean vapour density 9.6 g/m^3 ^(± (s.e.) 0.30).

### Live weight

There was a significant effect of treatment (P < 0.05) and treatment × time interaction (P < 0.05) for live weight. At d 2, on arrival in France, there was no difference (P > 0.05) in live weight between T (mean 231 ± (s.e.) 7.7 kg) and control animals (mean 242 ± (s.e.) 7.8 kg). On day 4, there was no difference (P > 0.05) in live weight between RT (mean 225 ± (s.e.) 6.90 and ULT (mean 227 ± (s.e.) 7.6 kg) animals, whereas RT and ULT animals had significantly lower (P < 0.05) live weight than control (mean 244 ± (s.e.) 7.6 kg). The mean percentage weight loss (± s.e.) for RT, ULT and control heifers on d 4 (at arrival in Spain) was 8.8 (0.78)%, 8.6 (0.67)% and 1.4 (0.41)% respectively, compared with d 0. The live weights of RT and ULT animals were not different (P > 0.05) from control on d 10 and d 34.

### Behaviour

The percentage (%) time spent standing by T animals during the road journey from the farm of origin to the ferry port was 91.2% (± (s.e.) 4.1); during the sea crossing (23 h) T animals spent 56% (± (s.e.) 7.4) of the time standing. Within 1 h of positioning the transporter on the roll-on roll-off ferry, 26 of the 40 animals were standing and during the last 3 h of the sea crossing no heifer was standing. During the 9 h road journey from the Ferry port to the lairage T animals spent 62% (± (s.e.) 5.6) of the time standing. During the 12 h rest period at the lairage in France there was no difference (P > 0.05) in the % time spent standing between RT and ULT animals; RT animals remaining on the transporter spent 77% (16.7 (± (s.e.)) of the time standing while ULT animals spent 64.4% (± (s.e.) 3.2). During the 23 h journey by road from France to Spain there was no difference (P > 0.05) in % standing time between RT and ULT animals; RT animals spent 45.9% (± (s.e.) 1.3) and ULT animals spent 56.8% (9.7 ± (s.e.) of the time standing. All T animals spent a significantly longer (P < 0.05)% time standing on the journey to the ferry compared with the % time spent standing during the sea crossing, during the journey by road from the ferry port to the lairage and on the journey by road from the lairage to the feedlot.

### Rectal temperature data

There was a significant effect of time (P < 0.001), and a treatment × time interaction (P < 0.001) and no effect (P > 0.05) of treatment, for rectal temperature measurements. Control animals had increased (P < 0.05) rectal body temperature on d 4 compared with d 0 (baseline values) (Table 1). RT animals remaining on the transporter in France had elevated rectal body temperature on d 4 (arrival in Spain) and on d 8 of the study compared with d 0. ULT animals that were unloaded at the lairage in France (d 2) had elevated body temperature (P < 0.05) at arrival in France and again on d 4 (upon arrival at the feedlot in Spain) compared with d 0 baseline. RT and ULT animals had lower (P < 0.05) rectal body temperature than control on d 3, d 6, whereas RT had higher (P < 0.05) body temperature on d 34 compared with control. One of the RT animals that remained on the transporter in France developed clinical signs of bovine respiratory disease on d 3, and presented with nasal discharge and cough (indications of upper respiratory tract disease), abnormal respiration (hyperpnoea; > 100 as an indication of lower respiratory tract disease), depression and elevated rectal temperature. The animal died on arrival at the Spanish feedlot on d 4.

**Table 1:** Changes in rectal body temperature (°C) in control and transported (RT and ULT) animals (n = 20 animals/treatment)

Variable	d 0 pre-transport	d 2 Arrival in French lairage	D 3 post-12 h lairage	d 4 arrival in Spain	d 6 post-transport	d 8 post-transport	d 10 post-transport	d 34 post-transport
**Control**	39.5 ± 0.15^ax^	39.5 ± 0.13^abx^	39.7 ± 0.15^abx^	40.3 ± 0.15^bx^	39.6 ± 0.14^abx^	39.5 ± 0.15^abx^	39.5 ± 0.59^abx^	39.0 ± 0.12^abx^
**RT**	39.2 ± 0.11^ax^	39.4 ± 0.14^abx^	39.0 ± 0.20^aby^	39.9 ± 0.19^bx^	39.0 ± 0.10^aby^	39.6 ± 0.12^bx^	39.6 ± 0.15^abx^	39.4 ± 0.12^aby^
**ULT**	39.2 ± 0.13^ax^	39.7 ± 0.17^bx^	39.1 ± 0.18^aby^	40.1 ± 0.19^bx^	39.1 ± 0.14^aby^	39.6 ± 0.18^abx^	39.7 ± 0.22^abx^	39.3 ± 0.12^abxy^

The values are expressed as mean (°C) ± s.e. Control = not transported; RT (transported animals that remained on the transporter at the French lairage for 12 h) and ULT animals transported (transported animals that were unloaded at the French lairage for a 12 h rest period) at a stocking density of 0.93 m^2 ^per animal;^a,b^Within a row, means not having a common superscript differ significantly (P < 0.05);^x,y^within a column at each sampling time point (day (d)), treatment means differ by P < 0.05. Data were analysed using SAS/STAT (9.1 (SAS Inst. Inc., Cary, NC, USA). The differences between means were tested using the Tukey-Kramer test for multiple comparisons.

### Inflammatory, adrenocortical and metabolic variables

There was a significant effect of treatment (P < 0.05) and treatment × time interaction (P < 0.05) for WBC number (Table 2). Control animals had lower (P < 0.05) WBC number on d 10 and greater (P < 0.05) number on d 34 compared with d 0 (baseline values). RT and ULT animals had greater (P < 0.05) WBC number on d 2 (upon arrival in France) to d 34 compared with d 0. WBC numbers were greater (P < 0.05) in RT and ULT animals compared with C animals from d 2 to d 34. There was no effect of treatment (P > 0.05) and time (P > 0.05), while treatment × time interaction was significant (P < 0.05) for neutrophil % and lymphocyte % (Table 2). Neutrophil % was lower (P < 0.05) in control animals on d 2, d 4, d 6 and d 34 compared with d 10 and was not different from baseline values (d 0) (Table 2). RT animals had greater (P < 0.05) neutrophil % on d 4 and d 8 than d 10 and were not different (P > 0.05) from d 0, whereas, ULT animals had lower (P < 0.05) neutrophil % on d 10 compared with C animals.

**Table 2:** Treatment effects on haematological variables pre and post-transport in control and transported (RT and ULT) animals (n = 20 animals/treatment)

Variable	d 0 pre-transport	d 2 Arrival in French lairage	D 3 post-12 h lairage	d 4 arrival in Spain	d 6 post-transport	d 8 post-transport	d 10 post-transport	d 34 post-transport
**White blood cell (WBC) number (1×10^3 ^μL)**								
**Control**	9.20 ± 0.57^ax^	8.20 ± 0.41^abx^	8.60 ± 0.54^abx^	7.90 ± 0.75^abx^	8.90 ± 0.75^abx^	7.81 ± 0.49^abx^	7.40 ± 0.44^bx^	9.66 ± 0.47^bx^
**RT**	10.10 ± 0.57^ax^	20.90 ± 2.30^by^	15.40 ± 1.99^by^	23.10 ± 1.43^by^	15.00 ± 1.43^by^	12.60 ± 1.01^by^	18.70 ± 1.74^by^	13.10 ± 1.22^by^
**ULT**	9.00 ± 0.59^ax^	16.70 ± 16.0^by^	14.0 ± 1.16^by^	17.80 ± 1.31^by^	15.60 ± 1.31^by^	14.10 ± 1.45^by^	16.10 ± 1.07^by^	11.14 ± 0.95^by^

**Neutrophil percentage (%)**								
**Control**	36.25 ± 2.70^abx^	32.95 ± 2.705^bx^	38.22 ± 3.10^abx^	33.20 ± 3.00^bx^	35.95 ± 2.85^bx^	38.10 ± 2.11^abx^	42.10 ± 2.42^ax^	32.44 ± 3.30^bx^
**RT**	38.00 ± 2.72^abx^	37.16 ± 2.76^abx^	39.20 ± 2.97^abx^	43.95 ± 3.01^bx^	37.16 ± 2.92^abx^	42.67 ± 2.21^bx^	33.79 ± 2.50^axy^	40.68 ± 3.10^abx^
**ULT**	37.30 ± 2.70^abx^	34.35 ± 2.70^abx^	37.78 ± 3.10^abx^	38.84 ± 3.00^abx^	34.55 ± 2.85^abx^	37.53 ± 2.25^abx^	31.47 ± 2.48^aby^	36.06 ± 3.23^abx^

**Lymphocyte percentage (%)**								
**Control**	62.35 ± 2.86^abx^	64.26 ± 2.74^abx^	60.11 ± 3.02^bx^	65.85 ± 2.92^abx^	62.85 ± 2.87^abx^	60.40 ± 2.05^abx^	57.30 ± 2.47^ax^	64.88 ± 14.14^abx^
**RT**	59.80 ± 2.90^abx^	58.63 ± 2.70^abx^	59.15 ± 2.92^abx^	53.89 ± 3.00^bx^	61.37 ± 2.14^abx^	54.94 ± 2.53^bx^	65.68 ± 3.10^axy^	58.42 ± 15.62^abx^
**ULT**	59.15 ± 2.80^abx^	62.05 ± 2.70^abx^	59.89 ± 3.01^abx^	59.00 ± 2.96^abx^	63.25 ± 2.86^abx^	60.82 ± 2.20^abx^	68.32 ± 2.53^aby^	62.82 ± 12.51^abx^

**Neutrophil:Lymphocyte ratio**								
**Control**	0.81 ± 0.13^abx^	0.70 ± 0.08^abx^	0.73 ± 0.10^abx^	1.00 ± 0.13^bx^	0.68 ± 0.09^abx^	0.82 ± 0.06^abx^	0.59 ± 0.08^ax^	0.89 ± 0.13^abx^
**RT**	0.70 ± 0.13^abx^	0.61 ± 0.08^abx^	0.79 ± 0.10^abx^	0.81 ± 0.13^bxy^	0.64 ± 0.08^abx^	0.65 ± 0.06^by^	0.49 ± 0.07^axy^	0.64 ± 0.13^abx^
**ULT**	0.69 ± 0.13^abx^	0.58 ± 0.78^abx^	0.70 ± 0.10^abx^	0.54 ± 0.13^aby^	0.63 ± 0.08^abx^	0.67 ± 0.06^abxy^	0.80 ± 0.07^aby^	0.57 ± 0.14^abx^

**Haematocrit (HCT) (%)**								
**Control**	39.49 ± 0.57^axy^	39.09 ± 0.41^abx^	38.32 ± 0.54^bx^	36.71 ± 0.66^bx^	36.51 ± 0.75^bx^	36.54 ± 0.49^bx^	35.39 ± 0.44^bx^	33.69 ± 0.47^bx^
**RT**	39.18 ± 0.67^ax^	34.41 ± 0.74^by^	32.17 ± 1.25^by^	32.26 ± 0.56^by^	31.36 ± 0.65^by^	29.75 ± 0.68^by^	29.78 ± 0.63^by^	28.09 ± 0.66^by^
**ULT**	40.23 ± 0.59^ay^	33.63 ± 0.90^by^	32.06 ± 1.16^by^	32.98 ± 1.69^by^	32.01 ± 1.31^by^	30.97 ± 1.45^by^	30.74 ± 1.07^by^	28.97 ± 0.95^by^

The values are expressed as mean ± s.e. Control = not transported; RT (transported animals that remained on the transporter at the French lairage for 12 h) and ULT animals transported (transported animals that were unloaded at the French lairage for a 12 h rest period) at a stocking density of 0.93 m^2 ^per animal;^a,b^Within a row, means not having a common superscript differ significantly (P < 0.05);^x,y^within a column at each sampling time point (day (d)), treatment means differ by P < 0.05. Data were analysed using SAS/STAT (9.1 (SAS Inst. Inc., Cary, NC, USA). The differences between means were tested using the Tukey-Kramer test for multiple comparisons.

Lymphocyte % was lower (P < 0.05) in control animals on d 10 compared with d 3 and were not different from baseline values (Table 2). RT animals had lower (P < 0.05) lymphocyte % on d 4 and d 8 compared with d 10. ULT animals had greater (P < 0.05) lymphocyte % than C on d 10.

There was no effect of treatment (P > 0.05) and time (P > 0.05), whereas the treatment × time interaction was significant (P < 0.05) for the N:L ratio. The N:L ratio was greater (P < 0.05) in control animals on d 4 compared with d 10 and were not different (P > 0.05) from baseline values (Table 2). RT animals had greater (P < 0.05) N:L ratio on d 4 and d 8 compared with d 10. ULT animals had lower (P < 0.05) N:L ratio on d 4, and greater (P < 0.05) N:L ratio on d 10 compared with C animals (Table 2).

There was a significant effect of treatment, time and treatment × time interaction (P < 0.05) for haematocrit % (Table 2). Control, RT and ULT animals had lower (P < 0.05) haematocrit % over time (d 2 to d 34) compared with d 0 (Table 2). RT and ULT animals had lower (P < 0.05) haematocrit % compared with C animals on d 2 to d 34.

There was a significant effect of treatment, time and treatment × time interaction (P < 0.05) for haemoglobin concentration (Table 3). Haemoglobin concentration was lower (P < 0.05) in C animals on d 4 to 34, in RT animals from d 3 to d 34, and in ULT animals from d 2 to d 34, compared with d 0. Haemoglobin concentration was lower (P < 0.05) in RT on d 3, d 6, d 8, d 10 and d 34 compared with C animals, whereas concentrations were lower (P < 0.05) in ULT animals on d 8 and d 34 compared with C animals.

**Table 3:** Treatment effects on haematological variables pre and post-transport in control and transported (RT and ULT) animals (n = 20 animals/treatment)

Variable	d 0 pre-transport	d 2 Arrival in French lairage	D 3 post-12 h lairage	d 4 arrival in Spain	d 6 post-transport	d 8 post-transport	d 10 post-transport	d 34 post-transport
**Haemoglobin (Hb) (g/dL)**								
**Control**	13.23 ± 0.17^ax^	13.15 ± 0.25^abx^	12.95 ± 0.20^abx^	12.38 ± 0.26^bx^	12.34 ± 0.25^bx^	12.24 ± 0.22^bx^	11.94 ± 0.26^bx^	11.46 ± 0.26^bx^
**RT**	13.14 ± 0.24^ax^	12.83 ± 0.25^abx^	11.91 ± 0.43^by^	12.05 ± 0.20^bx^	11.48 ± 0.22^by^	10.89 ± 0.22^by^	10.89 ± 0.21^by^	9.12 ± 0.23^by^
**ULT**	13.56 ± 0.32^ax^	12.49 ± 0.27^bx^	12.11 ± 0.25^bxy^	12.27 ± 0.26^bx^	12.01 ± 0.26^bxy^	11.41 ± 0.22^by^	11.37 ± 0.26^bxy^	9.42 ± 0.21^by^

**Red Blood Cell (RBC) number (×10^6^μL)**								
**Control**	10.51 ± 0.18^ax^	10.38 ± 0.18^abx^	10.05 ± 0.14^bx^	9.71 ± 0.16^bx^	9.74 ± 0.15^bx^	9.69 ± 0.16^bx^	9.42 ± 0.19^bx^	8.83 ± 0.27^bx^
**RT**	10.90 ± 0.21^axy^	11.75 ± 0.22^by^	10.93 ± 0.41^abxy^	11.10 ± 0.19^aby^	10.73 ± 0.24^aby^	10.31 ± 0.23^bxy^	10.13 ± 0.22^bxy^	7.31 ± 0.17^by^
**ULT**	11.28 ± 0.35^ay^	11.31 ± 0.25^aby^	11.10 ± 0.29^aby^	11.35 ± 0.28^aby^	11.01 ± 0.30^aby^	10.76 ± 0.26^by^	10.49 ± 0.30^by^	7.55 ± 0.16^by^

**Mean cell haemoglobin concentration (MCHC) (g/dL)**								
**Control**	33.50 ± 0.15^ax^	33.70 ± 0.12^abx^	33.80 ± 0.16^abx^	33.70 ± 0.16^abx^	33.80 ± 0.14^abx^	33.50 ± 0.12^abx^	33.80 ± 0.17^abx^	34.00 ± 0.18^bx^
**RT**	33.50 ± 0.18^ay^	37.30 ± 0.20^by^	37.10 ± 0.43^by^	37.34 ± 0.16^by^	36.90 ± 0.26^by^	36.90 ± 0.22^by^	36.60 ± 0.39^by^	32.20 ± 0.24^by^
**ULT**	33.70 ± 0.12^ay^	37.30 ± 0.32^by^	37.80 ± 0.25^by^	37.20 ± 0.20^by^	36.80 ± 0.24^by^	36.80 ± 0.22^by^	37.00 ± 0.30^by^	32.50 ± 0.17^by^

The values are expressed as mean ± s.e. Control = not transported; RT (transported animals that remained on the transporter at the French lairage for 12 h) and ULT animals transported (transported animals that were unloaded at the French lairage for a 12 h rest period) at a stocking density of 0.93 m^2 ^per animal;^a,b^Within a row, means not having a common superscript differ significantly (P < 0.05);^x,y^within a column at each sampling time point (day (d)), treatment means differ by P < 0.05. Data were analysed using SAS/STAT (9.1 (SAS Inst. Inc., Cary, NC, USA). The differences between means were tested using the Tukey-Kramer test for multiple comparisons.

There was a significant effect of treatment, time, and treatment × time interaction (P < 0.05) for RBC number and MCH concentration (Table 3). Control animals had lower (P < 0.05) RBC number on d 3 to d 34 than the pre-transport baseline. RT animals had greater (P < 0.05) RBC number on d 2, and lower (P < 0.05) RBC number on d 8 to d 34 compared with d 0. ULT animals had lower (P < 0.05) RBC number on d 8 to d 34 than d 0. RT animals had greater (P < 0.05) RBC number on d 2, d 4, d 6 and lower (P < 0.05) on d 34 compared with C animals. ULT animals had greater (P < 0.05) RBC number on d 0 to d 34 compared with C animals. MCH concentration was greater (P < 0.05) in control animals on d 34 than baseline. RT and ULT animals had greater (P < 0.05) MCH concentrations on d 2 to 10 and lower concentrations on d 34 than baseline values (Table 3). There was no difference (P > 0.05) between RT and ULT animals in MCH concentrations, however, values were greater (P < 0.05) than C animals on d 0 to d 34.

There was no effect of treatment, time or treatment × time interaction (P > 0.05) for concanavalin-A (Con-A) or phytohaemagglutinin A (PHA) induced interferon-(IFN)γ production concentration (data not shown).

There was no effect (P > 0.05) of treatment or time for plasma albumin concentrations, whereas, the treatment × time interaction was significant (P < 0.05) (Table 4). Control animals had lower (P < 0.05) albumin concentrations on d 4, d 6 and d 34 than d 0 values. RT animals had greater (P < 0.05) albumin concentrations on d 2 and d 3, and lower (P < 0.05) concentrations on d 6, d 8 and d 10 than d 0. RT animals had lower (P < 0.05) albumin concentrations on d 10 compared with C while ULT animals had lower (P < 0.05) albumin concentrations on d 2 compared with C.

**Table 4:** Treatment effects on metabolic variables pre and post-transport in control and transported (RT and ULT) animals (n = 20 animals/treatment)

Variable	d 0 pre-transport	d 2 Arrival in French lairage	D 3 post-12 h lairage	d 4 arrival in Spain	d 6 post-transport	d 8 post-transport	d 10 post-transport	d 34 post-transport
**Albumin (g/L)**								
**Control**	32.70 ± 1.37^ax^	34.90 ± 0.74^abx^	32.28 ± 0.89^abx^	32.10 ± 0.73^bx^	31.70 ± 0.70^bx^	32.10 ± 0.71^abx^	32.00 ± 0.73^abx^	30.50 ± 0.61^bx^
**RT**	32.40 ± 1.22^ax^	34.20 ± 0.53^bxy^	33.60 ± 0.58^bx^	33.20 ± 0.56^abx^	30.90 ± 0.37^bx^	30.90 ± 0.49^bx^	30.50 ± 0.4^by^	30.10 ± 1.76^abx^
**ULT**	34.25 ± 1.37^ax^	33.00 ± 0.76^by^	33.00 ± 0.72^abx^	33.40 ± 0.90^abx^	31.60 ± 0.73^bx^	31.30 ± 0.59^bx^	31.10 ± 0.63^bxy^	31.50 ± 0.62^bx^

**Globulin (g/L)**								
**Control**	41.10 ± 1.64^x^	39.40 ± 1.45^x^	40.30 ± 1.47^x^	41.30 ± 1.18^x^	41.80 ± 1.22^x^	41.20 ± 1.38^x^	43.30 ± 1.24^x^	43.00 ± 1.63^x^
**RT**	40.95 ± 1.25^ax^	41.76 ± 0.98^abx^	40.81 ± 1.00^abx^	43.79 ± 1.20^abx^	41.15 ± 1.47^abx^	44.82 ± 1.30^bx^	44.61 ± 1.24^bx^	44.37 ± 2.94^abx^
**ULT**	39.28 ± 1.47^ax^	39.28 ± 1.36^abx^	38.56 ± 1.41^abx^	40.99 ± 1.39^bx^	40.32 ± 1.21^abx^	42.85 ± 134^bx^	42.50 ± 1.28^bx^	43.71 ± 1.74^bx^

**Protein (g/L)**								
**Control**	73.82 ± 1.37^x^	72.41 ± 1.26^x^	72.57 ± 1.35^x^	73.42 ± 1.09^x^	73.48 ± 1.27^x^	73.29 ± 1.57^x^	75.35 ± 1.51^x^	73.56 ± 1.41^x^
**RT**	74.36 + 1.22^ax^	75.99 ± 0.91^aby^	74.48 ± 0.98^abx^	77.03 + 1.08^bx^	72.11 + 1.45^abx^	75.81 + 1.26^abx^	75.09 + 1.21^abx^	76.15 ± 4.23^abx^
**ULT**	73.09 ± 1.37^ax^	74.24 ± 1.14^abxy^	71.56 ± 1.13^abx^	74.38 ± 1.54^abx^	71.88 ± 1.16^abx^	74.17 ± 1.39^abx^	73.60 ± 1.22^abx^	75.18 ± 1.30^abx^

**ßeta-hydroxy butyrate (BHB) (mmol/L)**								
**Control**	0.29 ± 0.02^ax^	0.24 ± 0.01^bx^	0.26 ± 0.02^abx^	0.31 ± 0.02^abx^	0.31 ± 0.03^abx^	0.30 ± 0.02^abx^	0.22 ± 0.02^bx^	0.24 ± 0.02^bx^
**RT**	0.29 ± 0.02^ax^	0.28 ± 0.02^abx^	0.31 ± 0.02^abx^	0.35 ± 0.03^abx^	0.32 ± 0.02^abx^	0.26 ± 0.02^abx^	0.18 ± 0.02^bx^	0.19 ± 0.01^by^
**ULT**	0.33 ± 0.02^ax^	0.29 ± 0.03^abx^	0.32 ± 0.02^abx^	0.32 ± 0.02^abx^	0.35 ± 0.03^abx^	0.28 ± 0.03^abx^	0.23 ± 0.01^bx^	0.22 ± 0.01^bxy^

The values are expressed as mean ± s.e. Control = not transported; RT (transported animals that remained on the transporter at the French lairage for 12 h) and ULT animals transported (transported animals that were unloaded at the French lairage for a 12 h rest period) at a stocking density of 0.93 m^2 ^per animal;^a,b^Within a row, means not having a common superscript differ significantly (P < 0.05);^x,y^within a column at each sampling time point (day (d)), treatment means differ by P < 0.05. Data were analysed using SAS/STAT (9.1 (SAS Inst. Inc., Cary, NC, USA). The differences between means were tested using the Tukey-Kramer test for multiple comparisons.

There was no effect (P > 0.05) of treatment, an effect of time (P < 0.05) and treatment × time interaction (P < 0.05) for plasma globulin concentrations. RT animals had greater (P < 0.05) globulin concentrations on d 8 and d 10 compared with d 0, while ULT animals had greater (P < 0.05) concentrations on d 4, d 8, d 10 and d 34 compared with d 0 and were not different (P > 0.05) from C animals (Table 4).

There was no effect of treatment, or time (P > 0.05) for plasma protein concentrations, whereas there was a significant (P < 0.05) treatment × time interaction (Table 4). RT animals had greater (P < 0.05) protein concentrations on d 4 compared with d 0. There was no difference (P > 0.05) in protein concentrations between RT and ULT animals from d 2 to d 34 and concentrations were greater (P < 0.05) in RT animals than control on d 2.

There was an effect (P < 0.05) of time and no effect (P > 0.05) of treatment, or treatment × time interaction for (P > 0.05) for plasma ßHB concentrations. Control animals had lower (P < 0.05) βHB concentrations on d 2, d 10 and d 34 compared with d 0. RT and ULT animals had lower (P < 0.05) βHB concentrations on d 10 and d 34 compared with d 0, and RT animals had lower (P < 0.05) βHB concentrations on d 34 compared with control.

There was no effect of treatment or time (P > 0.05) for plasma NEFA concentrations, whereas there was a significant treatment × time interaction (P < 0.05) (Table 5). NEFA concentrations were lower (P < 0.05) in C animals on d 2, d 3, d 4, d 8 and d 34 compared with d 0. In RT and ULT animals, NEFA concentrations were lower (P < 0.05) on d 10 and d 34 compared with d 0. RT and ULT animals had greater (P < 0.05) NEFA concentrations on d 2, d 3, and d 4 and lower (P < 0.05) concentrations on d 10 and d 34 compared with C animals.

**Table 5:** Treatment effects on metabolic variables pre and post-transport in control and transported (RT and ULT) animals (n = 20 animals/treatment)

Variable	d 0 pre-transport	d 2 Arrival in French lairage	D 3 post-12 h lairage	d 4 arrival in Spain	d 6 post-transport	d 8 post-transport	d 10 post-transport	d 34 post-transport
**Non-esterified fatty acids (NEFA) (μmol/L)**								
**Control**	0.69 ± 0.07^ax^	0.38 ± 0.05^bx^	0.41 ± 0.05^bx^	0.45 ± 0.06^bx^	0.60 ± 0.06^abx^	0.51 ± 0.06^bx^	0.59 ± 0.05^abx^	0.43 ± 0.05^bx^
**RT**	0.67 ± 0.07^ax^	0.73 ± 0.08^aby^	0.79 ± 0.09^aby^	0.81 ± 0.10^aby^	0.71 ± 0.09^abx^	0.57 ± 0.08^abx^	0.29 ± 0.07^by^	0.07 ± 0.01^by^
**ULT**	0.64 ± 0.06^ax^	0.62 ± 0.07^aby^	0.61 ± 0.05^aby^	0.76 ± 0.07^aby^	0.66 ± 0.08^abx^	0.58 ± 0.07^bx^	0.43 ± 0.08^by^	0.06 ± 0.01^by^

**Urea (mmol/L)**								
**Control**	5.07 ± 0.42^ax^	3.97 ± 0.54^bx^	4.46 ± 0.61^abx^	4.73 ± 0.71^abx^	4.35 ± 0.61^bx^	3.27 ± 0.28^bx^	3.50 ± 0.32^bx^	3.06 ± 0.19^bx^
**RT**	4.42 ± 0.26^ax^	6.17 ± 0.65^by^	6.35 ± 0.82^by^	5.33 ± 0.31^by^	5.12 ± 0.47^aby^	4.56 ± 0.59^aby^	3.87 ± 0.47^aby^	4.92 ± 0.39^by^
**ULT**	4.57 ± 0.23^ax^	5.19 ± 0.22^by^	4.89 ± 0.22^aby^	4.56 ± 0.27^abxy^	4.62 ± 0.52^abxy^	4.37 ± 0.26^abxy^	4.64 ± 0.32^abxy^	5.12 ± 0.22^abxy^

**Glucose (mmol/L)**								
**Control**	4.88 ± 0.15^ax^	4.11 ± 0.08^bx^	4.19 ± 0.10^bx^	4.01 ± 0.11^bx^	4.24 ± 0.10^bx^	4.13 ± 0.10^bx^	4.08 ± 0.10^bx^	4.01 ± 0.08^bx^
**RT**	4.70 ± 0.23^ax^	4.53 ± 0.22^abxy^	4.43 ± 0.23^abxy^	4.43 ± 0.19^abxy^	4.13 ± 0.11^bxy^	4.26 ± 0.13^bx^	3.93 ± 0.23^bx^	4.96 ± 0.17^abx^
**ULT**	4.58 ± 0.18^ax^	5.02 ± 0.24^aby^	4.52 ± 0.13^aby^	4.34 ± 0.10^aby^	3.92 ± 0.07^by^	4.10 ± 0.08^bx^	4.05 ± 0.09^bx^	4.70 ± 0.13^abx^

The values are expressed as mean ± s.e. Control = not transported; RT (transported animals that remained on the transporter at the French lairage for 12 h) and ULT animals transported (transported animals that were unloaded at the French lairage for a 12 h rest period) at a stocking density of 0.93 m^2 ^per animal;^a,b^Within a row, means not having a common superscript differ significantly (P < 0.05);^x,y^within a column at each sampling time point (day (d)), treatment means differ by P < 0.05. Data were analysed using SAS/STAT (9.1 (SAS Inst. Inc., Cary, NC, USA). The differences between means were tested using the Tukey-Kramer test for multiple comparisons.

There was no effect (P > 0.05) of treatment for plasma urea concentrations, whereas there was a significant effect of time and treatment × time interaction (P < 0.05) (Table 5). Urea concentrations were lower (P < 0.05) in C animals on d 2, d 6, d 8, d 10 and d 34 compared with d 0. In RT animals, urea concentrations were greater (P < 0.05) on d 2, d 3, d 4 and d 34 while ULT animals had greater (P < 0.05) urea concentrations on d 2, compared with d 0. Urea concentrations were greater (P < 0.05) in RT animals from d 2 to d 34 compared with C.

There was no effect of treatment or time (P > 0.05) for plasma glucose concentrations, whereas there was a significant treatment × time interaction (P < 0.05) (Table 5). Glucose concentrations were lower (P < 0.05) in C animals from d 2 to d 34 compared with baseline values. In RT animals, glucose concentrations were lower (P < 0.05) on d 6, d 8 and d 10 and had normalised to d 0 values by d 34. ULT animals had lower (P < 0.05) glucose concentrations on d 6, d 8 and d 10 compared with d 0. ULT animals had greater (P < 0.05) glucose concentrations on d 2, d 3 and d 4, and lower values on d 6, compared with C.

There was no effect of treatment or time (P > 0.05) for plasma haptoglobin concentrations, whereas there was a significant treatment × time interaction (P < 0.05) (Table 6). Haptoglobin concentrations were lower (P < 0.05) in C animals than baseline on d 10 and d 34 (Table 6). RT and ULT animals had lower (P < 0.05) haptoglobin concentrations on d 34 compared with d 0.

**Table 6:** Treatment effects on haptoglobin, fibrinogen, creatine kinase (activity) and cortisol concentrations pre and post-transport in control and transported (RT and ULT) animals (n = 20 animals/treatment)

Variable	d 0 pre-transport	d 2 Arrival in French lairage	D 3 post-12 h lairage	d 4 arrival in Spain	d 6 post-transport	d 8 post-transport	d 10 post-transport	d 34 post-transport
**Haptoglobin (Hb binding capacity/L)**								
**Control**	1.49 ± 0.32^ax^	0.96 ± 0.22^abx^	0.84 ± 0.18^abx^	0.96 ± 0.16^abx^	0.92 ± 0.15^abx^	0.73 ± 0.12^abx^	0.55 ± 0.08^bx^	0.23 ± 0.04^bx^
**RT**	0.74 ± 0.22^ax^	0.84 ± 0.21^abx^	0.83 ± 0.20^abx^	1.25 ± 0.28^abx^	1.09 ± 0.25^abx^	0.94 ± 0.15^abx^	0.89 ± 0.12^aby^	0.28 ± 0.05^by^
**ULT**	0.81 ± 0.15^ax^	1.10 ± 0.18^abx^	1.08 ± 0.16^abx^	1.19 ± 0.18^bx^	1.02 ± 0.20^abx^	0.68 ± 0.15^abx^	0.68 ± 0.13^abxy^	0.39 ± 0.11^by^

**Fibrinogen (mg/dL)**								
**Control**	870 ± 99.3^ax^	989 ± 73.0^abx^	863 ± 64.0^abx^	1004 ± 67.0^abx^	853 ± 90.8^abx^	879 ± 55.3^abx^	849 ± 60.0^abx^	580 ± 36.5^bx^
**RT**	729 ± 79.4^ax^	835 ± 91.0^aby^	757 ± 74.9^abx^	923 ± 93.8^abx^	991 ± 109.0^abx^	746 ± 57.0^abx^	519 ± 35.0^by^	583 ± 46.0^bx^
**ULT**	785 ± 89.6^ax^	875 ± 70.8^abxy^	780 ± 63.7^abx^	912 ± 83.3^abx^	972 ± 78.7^abx^	658 ± 38.0^abx^	481 ± 38.2^by^	531 ± 48.6^bx^

**Creatine kinase (CK) (U/L)**								
**Control**	1391 ± 234.0^ax^	803 ± 176.0^bx^	679 ± 131.0^bx^	344 ± 49.0^bx^	434 ± 52.0^bx^	220 ± 19.6^bx^	2252 ± 28.2^bx^	580 ± 36.5^bx^
**RT**	812 ± 148.7^ay^	2058 ± 1630^abx^	531 ± 100.7^abx^	1215 ± 156.0^abx^	1222 ± 209.0^aby^	409 ± 105.0^by^	194 ± 28.0^bx^	583 ± 46.0^bx^
**ULT**	1379 ± 509.0^axy^	557 ± 101.0^bx^	578 ± 132.0^bx^	443 ± 81.0^bx^	1213 ± 199.0^aby^	375 ± 64.0^bxy^	340 ± 100.2^bx^	531 ± 48.6^bx^

**Cortisol (ng/mL)**								
**Control**	19.21 ± 3.11^abx^	9.26 ± 1.35^bx^	11.77 ± 1.59^abx^	12.77 ± 2.19^abx^	12.82 ± 1.40^abx^	12.84 ± 2.74^abx^	12.46 ± 1.44^abx^	19.74 ± 2.23^ax^
**RT**	20.68 ± 2.70^bx^	16.78 ± 4.91^abx^	9.56 ± 1.08^abx^	12.09 ± 2.04^abx^	12.01 ± 2.19^abx^	10.60 ± 1.58^abx^	8.11 ± 0.89^ax^	26.25 ± 3.76^bx^
**ULT**	19.49 ± 1.82^bx^	15.36 ± 1.47^abx^	9.41 ± 1.67^abx^	14.64 ± 1.74^bx^	8.98 ± 0.85^abx^	9.79 ± 0.74^abx^	10.44 ± 1.39^ax^	20.63 ± 2.53^bx^

The values are expressed as mean ± s.e. Control = not transported; RT (transported animals that remained on the transporter at the French lairage for 12 h) and ULT animals transported (transported animals that were unloaded at the French lairage for a 12 h rest period) at a stocking density of 0.93 m^2 ^per animal;^a,b^Within a row, means not having a common superscript differ significantly (P < 0.05);^x,y^within a column at each sampling time point (day (d)), treatment means differ by P < 0.05. Data were analysed using SAS/STAT (9.1 (SAS Inst. Inc., Cary, NC, USA). The differences between means were tested using the Tukey-Kramer test for multiple comparisons.

There was no effect of treatment or time (P > 0.05) for plasma fibrinogen concentrations, whereas there was a significant treatment × time interaction (P < 0.05) (Table 6). There was no change (P > 0.05) in fibrinogen concentrations from d 0 to d 10 in C animals, whereas concentrations were lower (P < 0.05) on d 34 than d 0. RT and ULT animals had lower (P < 0.05) concentrations of fibrinogen than baseline on d 10 and d 34 and concentrations were lower (P < 0.05) in RT on d 2, and in RT and ULT animals on d 10 compared with control.

CK activity was lower (P < 0.05) on d 2 to d 34 in control animals compared with baseline (Table 6) whereas, RT animals had lower (P < 0.05) CK activity on d 8, d 10 and d 34 than baseline. ULT animals had lower (P < 0.05) CK activity on d 2, d 3, d 4, d 8, d 10 and d 34 than baseline values. RT and ULT animals had greater (P < 0.05) CK activity on d 6 compared with control values whereas RT animals had greater (P < 0.05) CK activity than control on d 8 and were not different (P > 0.05) from ULT animals. There was no effect of treatment, time or treatment × time interaction (P > 0.05) for cortisol concentration (Table 6).

## Discussion

In the present study, the inflammatory, adrenocortical, metabolic and behavioural responses of weanling animals to transport were studied in 40 transported and 20 control heifers. The results of the study showed that transportation of weanling animals from Ireland to Spain affected live weight, haematological and some physiological variables of metabolism. Transportation can combine physical and psychological stressors, and weaning, loading and unloading, commingling of unfamiliar animals, loud noises, feed and water deprivation, extreme temperature, and the novelty of the transporter or new housing environment can be individually stressful, let alone in combination with each other [14,25-27]). In the present study, all animals were abruptly weaned and it is likely that the combined effects of weaning and transport associated with change in diet was implicit in some of the changes, in particular the live weight loss that was observed in the control animals. This decrease may be attributed to the management of the animals as they were weaned from their dams, removed from grazing pasture and fed *ad libitum *silage and 2 kg of concentrates from day 0. The loss in live weight recorded in the transported animals in the present study is in accordance with previously reported transport studies where live weight loss ranged from 3 to 11% [8-12,28]. Marahrens et al. [1] reported that loss of body weight in steers (-6.65%) coming from pasture was higher compared to bulls (-4.6%) during long distance transport but that animals recovered during lairage. In a review of cattle transport by road it was reported that approximately 3 to 11% loss of live weight occurs and that losses increase with increased journey times [6]. In the present study, the performance of the animals that remained on the transporter in France was not adversely affected post transport and was not different from the transported animals that were unloaded and lairaged in France.

Changes in the frequency and duration of basic behavioural patterns such as standing, lying and eating have often been used in the evaluation of the welfare status of animals [2,17]. The measurement of such behavioural patterns is appropriate where the environment or husbandry system may be hindering animals from eating or obtaining adequate rest, due to physical constraints, the actions of conspecifics or an increased level of stress. The behavioural responses of animals during transport, particularly lying and standing behaviour, are a useful measure of animal welfare during transport [6]. In the present study, the percentage of time spent standing was greater at the start of the journey and animals spent less time standing during the ferry journey and road journey to the feedlot. This finding is in accord with other groups [6] who observed that lying behaviour was more apparent during the latter stages of a journey when cattle were transported for 24 h. It was emphasized that the most stressful aspect of the transportation process for cattle was being confined on a moving vehicle and suggested that confinement on a stationary vehicle, loading/unloading and re-penning in a new environment are less stressful.

Rectal body temperature of the animals remaining on the transporter in France was elevated on days 4 and 8 of the study while animals that were unloaded at the lairage in France had elevated body temperature at the end of the 12 h rest period and at arrival in Spain on day 4. Of concern, was that one of the RT animals that remained on the transporter in France developed clinical signs of bovine respiratory disease on day 3, and presented with nasal discharge and indications of upper respiratory tract disease, abnormal respiration (hyperpnoea; > 100 as an indication of lower respiratory tract disease) and elevated rectal temperature. The animal died on arrival at the Spanish feedlot on day 4.

Several lines of evidence indicate that the psychological stress associated with transport, based on changes in heart rate and plasma cortisol concentrations, is generally highest during loading and pre-transport management of animals [13,14]. In the present study there was no change in plasma cortisol concentrations. In contrast with the present study, increased plasma cortisol concentrations, an indicator of hypothalamic-pituitary-adrenal (HPA) axis activation, have been reported in nearly all transportation studies of cattle as compared to pre-transportation baseline concentrations or those obtained from non-transported control [3,4,6,12,17,18,28-32]. Although circulating cortisol is the most predominant measure of stress studied in cattle, there are limitations to relying solely on this measure as an indicator of the extent of stress that an animal experiences. For example, a circadian rhythm dictates fluctuating concentrations of plasma cortisol regardless of exposure to a stressful event. In the present study, it was not possible to blood sample animals at the same time on each day of the blood sampling collection days (i.e. days 2 to 4) since animals were either lairaged for 12 h or in transit and it would not have been possible to access the animals at the standard blood collection time that we adhered to (8:00 GMT) for blood sampling. It is probable that if more frequent blood sampling was possible in the present study we might have been able to capture the cortisol response at the earlier stages of the transport. In a previous transport study of young bulls, we reported an acute increase in plasma cortisol in young bulls at 4.5 h relative to transport with cortisol concentrations reaching its lowest point at 14.25 h relative to transport [33]. Adaptation has been reported to take place during long distance transport as animals adapt to the novelty of transport while during short distance transport they don't habituate and may express acute (psychological) stress.

In the present study, haptoglobin concentration was lower in control animals than baseline on days 10 and 34 and all transported animals had lower haptoglobin concentrations than baseline on d 34, whereas concentrations were greater in RT and ULT than control on d 34. This latter finding may indicates a mild inflammatory response in transported animals compared with controls, although concentrations were lower on day 34 overall than baseline. Similarly, all transported animals had lower fibrinogen concentrations than baseline on d 10 and d 34. Results in the literature concerning changes in acute phase protein concentrations during transportation stress are variable. Serum haptoglobin was elevated in calves transported for 2 days in negative correlation with lymphocyte function [28]. In a separate study, transporting bulls at different stocking densities, plasma haptoglobin concentrations were unchanged, while plasma fibrinogen levels were reduced [8]. Plasma fibrinogen was greatly increased in a long distance transportation [26]. The results of the present study indicate that changes in acute phase protein concentrations were transient and were not significantly altered by transport. More investigation into acute phase proteins as biomarkers of transportation stress is necessary as transportation has been shown to both stimulate and suppress circulating concentrations. Arthington *et al*. [28] evaluated the effect of weaning and weaning plus transport in calves and found an increase in the concentration of haptoglobin in calves weaned but not in those weaned and transported, concluding that it is not necessary to have an inflammatory process to increase the concentration of this protein. Fibrinogen, ceruloplasmin, serum amyloid-A, and α-acid glycoprotein were assayed in the plasma of transported and commingled calves and found to be increased post-transportation; however, haptoglobin concentrations were higher in non transported versus transported calves [28].

In response to physical stress or exercise, the enzyme CK leaks from the sarcoplasm of muscle cells into the blood, due to increased permeability of the sarcolemma muscle cell membrane and therefore, elevated plasma CK activity is a useful indicator of muscular activity or muscle damage. Conversely, in the present study, CK activities were lower in all transported and control animals compared with their pre-transport baseline values. This would indicate that animals did not undergo physical activity as a consequence of the transport. Additionally, it also indicates good management and handling and that the conditions of the transport are more important than the transport itself.

The neutrophilia and lymphopenia, though transient, following transportation in this study are in agreement with previously reported findings following a variety of stressors, including transport stress [12,31-33]. White blood cell (WBC) numbers were greater in all transported animals from d 2 (at arrival in France) to d 34 while the control animals showed transient changes in WBC number. Furthermore, the changes in WBC number may suggest some form of dysregulation associated with mixing and assembly of the animals pre-transport.

Inexplicably, the haematocrit % declined across all treatments. It is probable that this decline may be related to the age of the animals and that the animals had *ad libitum *access to water and received the last feeding immediately before loading. All transported animals had lower haematocrit % than control from d 2 to d 34 and control animals had lower haematocrit % than baseline. It has been reported that raised haematocrit % following transport in association with higher erythrocyte counts in the circulation [11,12] indicates dehydration. Mormede et al. [34] reported that cattle were more susceptible to disease challenge in the days immediately following transportation. These observations have been rendered more concrete by a large body of work indicating that both intensity and duration of stressors may be important in bringing about changes in immunological functions [35]. In the present study, lymphocyte functional assays in terms of PHA-induced and Con-A induced IFN-γ production were used to assess immune function before and after transport. Induction of a proliferative response induced by antigen *in vitro *has been shown to be representative of cellular immunocompetence [36,37]. The present study showed that there were no major differences in IFN-γ concentrations after transport in animals that went through unloading and resting off the transporter at the lairage in France compared with animals that remained on the transporter. Lymphocytes play a critical role in host immunity to infection as they respond to infectious agents through production of antibodies, cytokines, and through specific T-cell mediated immune responses [38] and play a crucial role in the control of infection [39,40]. Furthermore, other investigators [16] observed a decrease of T lymphocytes in the peripheral blood of calves after transportation, however, evaluation of the T lymphocyte subpopulations was not performed. More recently, alterations in peripheral blood lymphocyte subsets in transported calves with increased cortisol and catecholamine concentrations were reported [41] and transport induced a significant reduction in peripheral blood lymphocyte subsets detected by a panel of mAbs which were no longer present at 24 h after arrival.

In the present study, the changes in metabolic variables were similar to previously reported changes after transport [8,9]. These observations have been rendered more concrete by a large body of research indicating that metabolic variables are useful indicators in the diagnosis and prognosis of pathological states [42]. In the present study, the RT and ULT animals showed similar responses to transport. There was no difference in protein concentrations between RT and ULT animals from d 2 to d 34. Glucose concentrations were lower in control animals from d 2 to d 34 compared with baseline values. In RT animals, glucose concentrations were lower on d 6, d 8 and d 10 and had normalised to d 0 values by d 34. ULT animals had lower glucose concentrations on d 6, d 8 and d 10 compared with d 0. Changes in the circulating concentrations of biological variables are often used to study the impact of treatments on metabolism. Interestingly, ßHB concentrations were lower than baseline in control and all transported animals on d 10 and d 34. Previous data in the literature indicate that the central nervous system plays an important role in the regulation of hepatic glucose and lipid metabolism via the sympathetic nervous system and metabolic hormones [43]. ßHB is a key indicator of hepatic ketogenesis and is the primary ketone body found in blood. Prolonged fasting has been shown to increase lipid catabolism resulting in higher blood concentrations of βHB. RT and ULT animals had lower non-esterified fatty acid (NEFA) and βeta-hydroxy-butyrate (ßHB) concentrations on d 10 and d 34 compared with d 0. Urea concentrations were lower in control animals on d 2, d 6, d 8, d 10 and d 34 compared with d 0. In RT animals, urea concentrations were greater on d 2, d 3, d 4 and d 34 while ULT animals had greater urea concentrations on d 2, compared with d 0. It is possible that the changes reported in NEFA, ßHB and urea concentrations in this study, may be related to the effects of weaning in combination with transport, dietary change and the journey duration.

## Conclusions

In conclusion, the results from this study show that animals undergoing transportation by road and sea, followed by road, at a spatial allowance of 0.93 m^2 ^showed inflammatory, adrenocortical, metabolic and behavioural responses that were within normal referenced ranges [44-46]. Within the conditions of the present study, the performance of the animals that remained on the transporter during the 12 h lairage period in France was not different post-transport from the transported animals that were unloaded and lairaged in France. It is concluded that there were no meaningful differences between unloaded animals and animals that remained on the transporter during the 12 h lairage period.

## Methods

### Care of animals

All procedures were conducted under experimental license from the Irish Department of Health in accordance with the Cruelty to Animals Act 1876, and the European Communities (Amendment of Cruelty to Animals Act, 1876) Regulations, 1994.

### Transport vehicle and environmental conditions

The study was conducted in November 2003. At the end of the grazing season, 60 Charolais × beef heifers were weaned (mean live weight 245, s.e. 4.3 kg) (mean weaning age 219, s.e. 4.9 days) on day 0 from their dams on farm of origin, were socially mixed, re-grouped and assigned by live weight to two treatments, control (C ) (n = 20) (mean live weight 247, s.e. 7.2 kg) and transport (T) (n = 40) (mean live weight 246, s.e. 5.4 kg). On the morning of the journey, the 60 weaned animals were blood sampled (day 0) by jugular venepuncture to provide baseline inflammatory, adrenocortical and metabolic values on the farm of origin. Twenty C animals remained in Ireland and were not transported. Forty T animals were loaded at 18:00, onto the lower deck of an air suspension articulated transporter (total area = 30.96 m^2^) which was divided into 4 fan-ventilated pens at a stocking density of 0.93 m^2 ^per animal, and transported by road for 3 h to the ferry port. The four pens on the transporter were bedded with a deep bed of cereal straw and water was available through nipple drinkers. The truck was then driven on to the ferry by an experienced driver. The ferry journey took approximately 23 h. The average speed during the sailing ranged from 11 to 13.5 knots/hr, the wind/force ranged from SE/5 - SE/6, and the ambient temperature ranged from 8 to 11°C. The temperature of the transporter during the sea crossing ranged from 13 to 15°C. On arrival at the French port, Cherbourg, T animals were transported by road for 9 h to a French lairage. Upon arrival at the French lairage, two pens of T animals (n = 10 animals/pen) situated in the rear part of the transporter were unloaded (ULT) and rested for 12 h in the French lairage and 20 animals rested (RT) on the transporter. The animals from the respective pens were not mixed. All animals had access to hay and water. ULT animals were housed at a spatial allowance of 2 m^2^/animal at the lairage in a straw bedded shed. After the 12 h rest period, the straw bedding was renewed on the transporter, the ULT animals were re-loaded and maintained in their original pens on the transporter. The total time taken to complete the remaining stage of the transport journey by road from France to Spain was 23 h (which included 9 h travel (start time 17:00 GMT), 7 h compulsory rest (on the transporter) and a further 7 h travel by road. The journey was made by the same driver and involved a combination of road surfaces ranging from motorways, secondary roads to small country lanes. All T animals were blood sampled at 8:00 GMT prior to transport (d 0), on arrival (03:00 GMT) in the French lairage (d 2) after 12 h rest (15:00 GMT) in the French lairage (d 3), on arrival (16:00 GMT) at the feedlot in Spain (d 4) and at 8:00 GMT on d 6, 8, 10 and 34. Control animals were blood sampled at the same times (GMT) as T animals. The time taken to blood sample each animal was approximately 2 to 3 min. Animals were weighed on d 0, 3, 4, 10 and 34 of the study. The control animals were housed (d 0) in a straw bedded shed on the farm of origin at a space allowance of 4 m^2^/head and had *ad libitum *access to grass silage and 2 kg of a barley/soybean concentrate. Control animals were weighed on d 0, 3, 4, 10 and 34 of the study using identical precision platform scales to the one used for the T animals (O'Donovan Engineering Co. Ltd, Cork, Ireland). The transporter was fitted with sensors (horizontally to the direction of travel) above the animals, for measuring ambient temperature (°C), relative humidity (RH; %), carbon dioxide (CO_2; _ppm), air velocity (m/s) and vapour density (g/m^3^) continuously during transport. The ambient temperature and relative humidity during transport were recorded continuously using TinyTalk dataloggers (Radionics, Dublin, Ireland). Environmental measurements on the transporter including gas (CO_2_), relative humidity (RH) and temperature were recorded using QRae (Shawcity Ltd., UK), Testo 175 and Testo 445 portable multifunction probes (Testo UK, Ltd).

### Animal diets and composition

Control animals had *ad libitum *access to grass silage (in vitro DM digestibility = 755 g/kg), supplemented with 2.0 kg (as fed) barley/soybean concentrate (DM 843 g/kg; crude protein = 115 g/kg DM) per animal per day on the farm of origin. During the rest period in France, the RT and ULT animals had *ad libitum *access to hay (DM 527 g/kg; ash 83.0 g/kg; acid detergent fibre (ADF) 342 g/kg) and water.

The animals in Spain were fed an *ad libitum *finishing diet, consisting of concentrate diet (DM 875 g/kg; crude protein 153 g/kg DM) and straw (DM 899 g/kg; crude protein 45 g/kg DM). All animals had free access to water available through nipple drinkers.

### Body (rectal) temperature

In parallel with the blood sampling collections, rectal body temperature was taken prior to transport (day (d) 0), on arrival in the French lairage (d 2), after 12 h rest in the lairage (d 3), on arrival at the feedlot in Spain (d 4) and on d 6, 8, 10 and 34 of the study. The rectal body temperature was monitored using a digital thermometer (Jorgen Kruuse A/S; Model VT-801BWC Lot No 0701). Animals were observed for general illness and specific clinical signs of respiratory tract disease.

### Behaviour

Lying and standing behaviour of all animals on the transporter was continuously video-recorded using black-white cameras (Eneo, Germany) with built in 12 watt infra red lighting positioned in the 4 individual pens of the transporter. The cameras recorded 19-25 frames/sec and images were transferred to a personal computer using a multiplex card manufactured by CCTV (UK). The animals were observed by instantaneous scan sampling and the interval between scans was 10 min. A count of the total number of occurrences of each behaviour was made for each scan time point. For presentation purposes, the percentage time values were calculated from the total count data for lying and standing behaviours. As the animals were subjected to continuous recordings, the count data was expressed as percentage time.

### Assay procedures for inflammatory, adrenocortical and metabolic variables

Heparinised blood samples (4 × 9 mL) were collected by jugular venepuncture and the plasma was separated by centrifugation at 1,600 × g at 8°C for 15 min, and subsequently stored at -20°C until assayed. Plasma albumin, urea, globulin, total protein, β-hydroxybutyrate (βHB), and glucose concentrations, and creatine kinase (CK) activity were measured on an automatic analyser (Olympus AU 400, Japan).

Plasma haptoglobin was determined as the hemoglobin (HB) binding capacity using an assay kit (Tridelta Development Ltd., Wicklow, Ireland), and was measured on an automatic analyzer (spACE, Alfa Wassermann, Inc., West Caldwell, NJ, USA). Plasma fibrinogen was determined using a commercial kit (Roche-Boerhinger, Mannheim, Germany) adapted for bovine plasma [47].

Red blood cell (RBC) number, white blood cell (WBC) number, differential WBC (percentage lymphocyte and neutrophil), packed cell volume (haematocrit), haemoglobin (Hb) concentration, mean corpuscular haemoglobin concentration (MCHC) and platelet numbers were determined for unclotted (EDTA-treated) whole-blood samples (5 mL) with an automated cell counter (Celltac MEK-6108K; Nihon-Kohdon, Tokyo, Japan) within 1 h of blood sampling. A standard bovine haematology control was measured to control for instrument variation. Thin blood smears were also prepared on glass slides and stained using the haematology 3-step stain for differential WBC number (Accralab, Biochemical Sciences; Fisher Scientific Company, Middletown, VA).

Blood samples for interferon-γ (IFN-γ) determination were collected by jugular venepuncture into aseptic vacutainer tubes containing lithium heparin and the stimulated lymphocyte production of IFN-γ was determined following whole blood culture. The lymphocyte production of IFN-γ was determined [48] in duplicate following stimulation *in vitro *for 24 h at 37°C and in an atmosphere of 5% CO_2_, with either phosphate buffered saline alone (PBS), phytohaemagglutinin A (PHA; 20 μg/1.5 mL blood), or concanavalin A (Con-A; 20 μg/1.5 mL blood) in whole blood culture; the IFN-γ concentration in the harvested plasma samples was measured using a specific ELISA procedure (CSL, Biosciences, Parkville, Victoria, Australia).

Plasma was harvested from heparin anti-coagulated blood following its centrifugation at 1600 × g at 4°C for 15 minutes and stored at -80°C until subsequent cortisol analysis. The *in vitro *Con-A and PHA stimulated production of IFN-γ was calculated by subtracting the absorbance at 450 nm of wells that received PBS alone from wells that received Con-A or PHA, respectively. Commercial RIA kits were used to determine the plasma concentrations of cortisol (Corti-cote, ICN Pharmaceuticals, Orangeburg, NY; validated by Fisher *et al *[49]. The intraassay CV (n = 6 per assay) for samples containing 5.3, 17.9 and 56.7 ng of cortisol/mL were 16.3, 9.5 and 9.7%, respectively. The interassay CV (n = 15) for the same samples were 15.8, 11.9 and 13.8%, respectively.

### Statistical analyses

Data were analysed using SAS/STAT (9.1 (SAS Inst. Inc., Cary, NC, USA) repeated measures. Inflammatory, adrenocortical, metabolic and live weight data were analysed using the repeated measures procedure in PROC MIXED procedure of SAS with an unstructured covariance matrix within animal. Sampling time, treatment and interactions were listed in the model statement. A probability of P < 0.05 was chosen as the level of significance for the statistical tests. Measurements for WBC number, MCHC, albumin, glucose, NEFA, urea, haptoglobin, fibrinogen concentrations and CK activity were shown by Levene and Shapiro-Wilks tests to be non-normal, and data were log transformed prior to statistical analyses. Differences between means were determined using the Tukey-Kramer test for multiple comparisons, and the associated P-values presented were derived from the statistical analysis of the data using the model described above. Percentage time values were calculated from the total count data for lying and standing behaviours. A count of the total number of occurrences of lying and standing behaviour was made for each scan time point. As the animals were subjected to continuous recordings, the count data was expressed as percentage time. The behavioural data were analysed using the Mann-Whitney U test for non-parametric data [50]. Wilcoxon Matched Pairs Signed Rank test was used to test for within treatment comparisons.

## Competing interests

The authors declare that they have no competing interests.

## Authors' contributions

BE designed the study. BE and MM performed the experiments. BE analyzed the data and prepared the manuscript. All authors read and approved the final manuscript.
